# The Soul’s Wisdom: Stories of Living and Dying

**DOI:** 10.3747/co.v15i0.272

**Published:** 2008-08

**Authors:** M.L.S. Vachon

**Affiliations:** Departments of Psychiatry and Public Health Sciences, University of Toronto, Toronto, Ontario, Canada

**Keywords:** Spirituality

## Abstract

Cancer can lead to spiritual transformation, which can be seen as a form of alchemy. During this process, patients, family members, and even professional caregivers can find themselves having spiritual experiences that go beyond any they had previously encountered. This paper provides qualitative descriptions of the “Field” or “Soul Wisdom” experienced by patients and caregivers.

